# Racial and Ethnic Disparities in Cannabis Use Among U.S. Youth Who Use Tobacco: Findings From the Population Assessment of Tobacco and Health Study

**DOI:** 10.1016/j.focus.2025.100392

**Published:** 2025-07-01

**Authors:** Allison A. Temourian, Pamela M. Ling, Vuong V. Do, Nhung Nguyen

**Affiliations:** 1 Center for Tobacco Control Research and Education, Cardiovascular Research Institute, University of California San Francisco, San Francisco, California; 2 Division of General Internal Medicine, Department of Medicine, University of California San Francisco, San Francisco, California

**Keywords:** Adolescent, cannabis, tobacco, racial disparities

## Abstract

**Introduction:**

Limited research on youth tobacco use addresses cannabis use across different tobacco products and racial/ethnic subgroups. This study examines these patterns of cannabis use among youth who use tobacco using 6 waves of nationally representative data.

**Methods:**

This study analyzed the Population Assessment of Tobacco and Health Study data Waves 1–6 (during 2013–2021) in 2024. The authors conducted cross-sectional weighted analyses for each wave. The authors estimated past 30-day use of cannabis among youth who reported past 30-day use of any tobacco, combustible tobacco, and e-cigarette for the total sample and for each racial and ethnic group (Hispanic, non-Hispanic White, non-Hispanic Black, and non-Hispanic other).

**Results:**

Across 6 waves, cannabis-use prevalence was over 36% among youth who used any tobacco product, over 40% among those who used combustible tobacco, and 36% among those who used e-cigarettes. There were racial/ethnic disparities in cannabis use among youth who used e-cigarettes, with non-Hispanic Black, non-Hispanic other, and Hispanic youth reporting the highest cannabis-use prevalence. There were no significant racial/ethnic differences in cannabis use among youth who used combustible tobacco.

**Conclusions:**

Cannabis use is highly prevalent among youth who use tobacco, with significant disparities by tobacco product and race/ethnicity. The disproportionately high prevalence of cannabis use among non-White youth highlights the need for tailored interventions to address these disparities in cannabis and tobacco use among youth.

## Highlights

• Cannabis-use prevalence among youth using tobacco ranged from 36% to 61%.
• Racial/ethnic differences in co-use were observed among youth who use e-cigarettes.
• Non-Hispanic Black, non-Hispanic other race/ethnicity, and Hispanic youth had the highest cannabis-use prevalence.

## INTRODUCTION

Tobacco and cannabis remain the most common substances used among U.S. youth.^1^ In 2024, 10.1% and 13.4% of high-school students reported past 30-day use of tobacco^2^ and cannabis,^3^ respectively. Use of either tobacco or cannabis during adolescence has been linked to negative health effects, including poorer cognitive functioning,^4^^,^^5^ mental health,^6^^,^^7^ and increased risk of respiratory illness.^8^^,^^9^ Using tobacco and cannabis concurrently (co-use) may lead to additive health effects (e.g., mental health distress), increased use and dependence, and poorer cessation outcomes.^10^, ^11^, ^12^, ^13^ Population-based data on cannabis use among youth who use tobacco are necessary to inform efforts to mitigate harms related to these substances.

Data on youth’s co-use are lacking. A study examining youth co-use reported that 47% of youth who used cigarettes and 40% of youth who used e-cigarettes also used cannabis in 2015.^14^ A more recent study found that 3.4% of youth reported co-use in the past 30 days.^10^ In addition, a recent study showed racial/ethnic disparities in tobacco and cannabis use and co-use, with Black and Hispanic youth reporting more cannabis-only but less tobacco-only use than White peers; American Indian or Alaska Native youth had more co-use, whereas Asian youth used both substances less.^13^ Although prior studies documented evidence of racial/ethnic disparities in cannabis and tobacco co-use,^14^^,^^15^ little is known about how cannabis-use prevalence varies by type of tobacco product used within each racial and ethnic subgroup.

In the past decade, the tobacco and cannabis industries have had major changes, including the rapid rise in popularity of e-cigarettes^16^ and the expanded legalization of recreational cannabis across the U.S.^17^ These changes significantly influenced youth access,^18^ product availability,^19^ and perceptions of harm.^20^ Given the changing contexts of tobacco product landscape and cannabis legalization, updated national estimates are warranted. This study estimated the population-based prevalence of youth cannabis use by type of tobacco product used across racial and ethnic subgroups using the Population Assessment of Tobacco and Health (PATH) Study data from 2013 to 2021.

## METHODS

### Study Sample

The PATH Study is an ongoing, nationally representative, longitudinal cohort study of tobacco-use behaviors, attitudes, and beliefs and tobacco-related health outcomes among youth (aged 12–17 years) and adults (≥18 years in the U.S).^21^ Participants are noninstitutionalized and recruited through a 4-stage stratified area probability sampling design. The PATH Study is conducted by Westat and approved by their IRB, which waived the need for informed consent to examine public-use files. Additional information regarding PATH data (including data files) can be downloaded directly from the PATH website.^22^

This study used Waves 1–6 of youth data (public-use files). The total sample and data collection period for each wave were *n*=13,651 (September 2013–December 2014) for Wave 1; *n*=12,172 (October 2014–October 2015) for Wave 2; *n*=11,814 (October 2015–October 2016) for Wave 3; *n*=14,798 (December 2016–January 2018) for Wave 4; *n*=12,098 (December 2018–November 2019) for Wave 5; and *n*=5,652 (March 2021–November 2021) for Wave 6. Race/ethnicity samples in each wave can be found in Appendix Table 1 (available online).

### Measures

Current use of tobacco was self-reported use of any tobacco product in the past 30 days (yes/no), including e-cigarettes and combustible products (cigarettes, cigars, cigarillos, filtered cigars, pipes, hookah, bidi, kretek, and blunt).

Current use of cannabis was self-reported use of any cannabis/marijuana product in the past 30 days (yes/no), including smoking products (e.g., joint, bong, blunt), vaping cannabis liquids/oils, or cannabis used in other ways.

Race and ethnicity were self-reported and categorized as Hispanic/Latinx, non-Hispanic (NH) White, NH Black, and NH other (i.e., American Indian or Alaska Native, Asian, Native Hawaiian or other Pacific Islander, and multiracial).^21^

### Statistical Analysis

Within each wave, cannabis-use prevalence was compared across 3 tobacco product types (any tobacco, e-cigarettes, and combustible). Then, within each type of tobacco-use subgroup, the authors compared cannabis-use prevalence within racial/ethnic groups using chi-square tests. The authors stacked the 6 waves of data and conducted cross-sectional weighted analyses for each wave.^23^ This study did not analyze trends over time owing to inconsistencies in survey administration and measures. Analyses were weighted to obtain nationally representative estimates, and variances were estimated using balanced repeated replication, with Fay’s adjustment set to 0.3.^23^ All hypothesis tests were 2 sided, and *p*≤0.05 was considered statistically significant. Data were analyzed in 2024 using STATA 18.0.^24^^,^^25^

## RESULTS

Regarding tobacco- and cannabis-use patterns, 5.3% of participants (95% CI=5.1%, 5.6%) reported tobacco use only, 3.1% (95% CI=2.9%, 3.3%) reported cannabis use only, and 3.2% (95% CI=3.0%, 3.4%) reported using both tobacco and cannabis in the past 30 days (co-use). Among NH White youth, 6.8% (95% CI=6.3%, 7.2%) reported tobacco use only, 2.9% (95% CI=2.6%, 3.1%) reported cannabis use only, and 3.6% (95% CI=3.4%, 3.9%) reported co-use. Among NH Black youth, 3.3% (95% CI=2.7%, 3.9%) reported tobacco use only, 3.6% (95% CI=3.1%, 4.2%) reported cannabis use only, and 2.5% (95% CI=2.0%, 2.9%) reported co-use. Among NH other race/ethnicity youth, 3.8% (95% CI=3.3%, 4.4%) reported tobacco use only, 2.7% (95% CI=2.3%, 3.2%) reported cannabis use only, and 2.8% (95% CI=2.3%, 3.5%) reported co-use. Among Hispanic/Latinx youth, 4.1% (95% CI=3.7%, 4.6%) reported tobacco use only, 3.5% (95% CI=3.1%, 3.8%) reported cannabis use only, and 2.7% (95% CI=2.4%, 3.1%) reported co-use. Prevalence rates of any tobacco or cannabis use can be found in Appendix Table 2 (available online).

**Figure 1:**
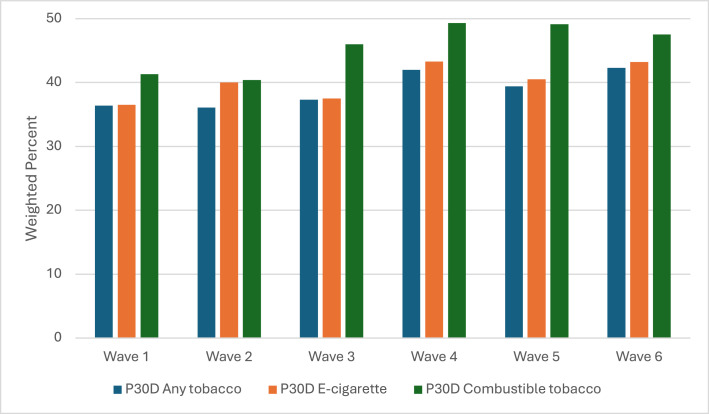
Cannabis-use prevalence among youth who use tobacco. P30D, past 30 day.

Across the 6 waves, over 36% of youth who reported currently using any tobacco also reported using cannabis (Figure 1), with the highest prevalence of cannabis use in Wave 6 (42.3%; 95% CI=36.6%, 48.1%) (Table 1). Of those who used e-cigarettes, over 40% also used cannabis, with the highest prevalence in Waves 4 (43.3%; 95% CI=39.1%, 47.5%) and 6 (43.2%; 95% CI=37.3%, 49.3%). The prevalence of cannabis use among those who used combustible tobacco was slightly higher than among those who used e-cigarettes, with the highest prevalence in Waves 4 (49.3%; 95% CI=45.5%, 53.1%) and 5 (49.1%; 95% CI=42.8%, 55.4%).

**Table 1:** Prevalence of Cannabis Use Among Youth Who Use Tobacco (Weighted % and 95% CI)

Wave	Total	NH White	NH Black	NH other	Hispanic	*p*-value
Any tobacco users						
1	36.4 (33.5, 39.4)	32.3 (28.6, 36.4)	48.5 (40.1, 56.9)	45.3 (33.4, 57.8)	39.6 (33.1, 46.4)	0.7190
2	36.1 (32.5, 39.8)	33.9 (29.5, 38.6)	40.6 (28.5, 53.9)	42.1 (30.8, 54.3)	38.6 (32.2, 45.5)	0.0500
3	37.3 (34.4, 40.3)	36.8 (33.0, 40.7)	33.6 (24.5, 44.1)	51.2 (39.5, 62.7)	37.1 (29.9, 44.9)	0.0020
4	42.0 (39.0, 45.0)	39.6 (35.6, 43.7)	51.1 (40.0, 62.1)	44.6 (34.2, 55.4)	44.5 (38.4, 50.8)	0.0222
5	39.4 (36.6, 42.2)	36.8 (33.5, 40.3)	51.6 (39.2, 63.8)	41.2 (31.3, 51.9)	42.0 (36.3, 47.9)	0.0003
6	42.3 (36.6, 48.1)	41.7 (34.6, 49.3)	38.3 (22.1, 57.5)	34.9 (18.3, 56.3)	46.9 (33.1, 61.2)	0.0626
E-cigarette users						
1	36.5 (31.9, 41.3)	36.4 (31.0, 42.0)	31.5 (19.2, 47.1)	43.0 (28.4, 59.1)	35.8 (25.3, 47.9)	0.0107
2	40.0 (34.5, 45.6)	40.4 (33.9, 47.4)	32.6 (13.1, 60.8)	41.8 (26.8, 58.6)	38.1 (28.6, 48.7)	0.0001
3	37.5 (33.3, 41.8)	35.1 (29.9, 40.7)	44.0 (28.9, 60.3)	59.0 (44.1, 72.4)	37.1 (28.0, 47.1)	0.0605
4	43.3 (39.1, 47.5)	41.1 (35.5, 46.9)	60.0 (35.5, 80.4)	45.1 (31.0, 60.0)	46.9 (37.5, 56.6)	0.0003
5	40.5 (37.8, 43.3)	38.8 (35.3, 42.5)	48.6 (33.7, 63.8)	39.6 (29.5, 50.6)	43.9 (37.5, 50.4)	<0.001
6	43.2 (37.3, 49.3)	42.5 (35.0, 50.3)	43.0 (22.7, 66.0)	40.1 (23.2, 59.7)	46.9 (32.6, 61.7)	0.0413
Combustible tobacco users						
1	41.3 (38.2, 44.4)	35.5 (31.1, 40.1)	54.7 (45.1, 64.0)	55.5 (41.3, 68.8)	46.1 (38.4, 53.9)	0.7254
2	40.4 (36.1, 44.9)	37.0 (31.8, 42.6)	44.1 (30.6, 58.6)	47.6 (34.3, 61.3)	46.2 (37.8, 54.9)	0.3482
3	46.0 (41.7, 50.4)	47.5 (42.4, 52.6)	32.0 (21.3, 45.2)	56.0 (38.9, 71.7)	46.4 (35.0, 58.2)	0.1065
4	49.3 (45.5, 53.1)	48.5 (43.0, 54.0)	51.7 (39.9, 63.3)	52.9 (41.5, 63.9)	49.2 (41.7, 56.8)	0.7606
5	49.1 (42.8, 55.4)	48.0 (40.2, 55.8)	57.2 (40.8, 72.2)	49.3 (30.7, 68.1)	46.5 (35.7, 57.6)	0.1499
6	47.5 (36.0, 59.3)	46.3 (31.2, 62.2)	42.4 (15.6, 74.5)	25.5 (2.5, 82.2)	61.3 (35.7, 81.9)	0.1705

*Note: p*-value refers to a significant chi-square estimate of differences between racial/ethnic group prevalence of cannabis use by tobacco use status.

NH, non-Hispanic.

**Figure 2:**
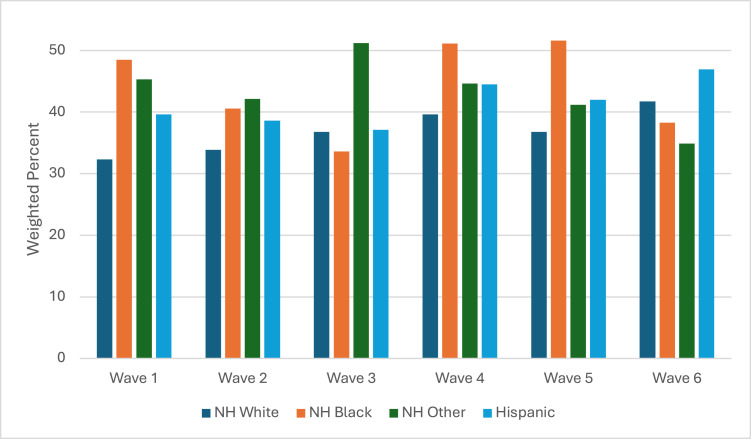
Racial/ethnic differences in cannabis-use prevalence among youth who use any tobacco. NH, non-Hispanic.

Cannabis-use prevalence among youth who use any tobacco product was significantly different by race/ethnicity, except in Waves 1 and 6 (Figure 2 and Table 1). NH Black youth had the highest prevalence of cannabis use in Wave 1 (48.5%; 95% CI=40.1%, 56.9%), Wave 4 (51.1%; 95% CI=40%, 62.1%), and Wave 5 (51.6%; 95% CI=39.2%, 63.8%). NH other race/ethnicity youth had the highest prevalence of using cannabis in Wave 2 (42.1%; 95% CI=30.8%, 54.3%) and Wave 3 (51.2%; 95% CI=39.5%, 62.7%). Hispanic youth had the highest prevalence of using cannabis in Wave 6 (46.9%; 95% CI=33.1%, 61.2%).

**Figure 3:**
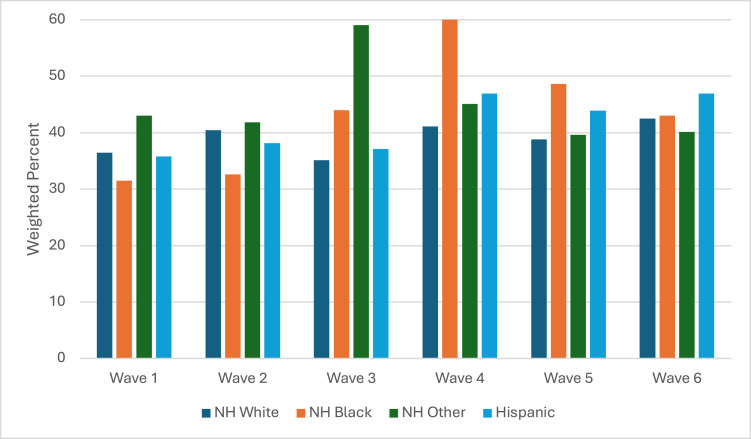
Racial/ethnic differences in cannabis-use prevalence among youth who use e-cigarettes. NH, non-Hispanic.

There were significant racial/ethnic differences in cannabis use among youth who use e-cigarettes for all waves, except in Wave 3 (Figure 3 and Table 1). NH Black youth who used e-cigarettes had the highest prevalence of using cannabis in Waves 4 (60.0%; 95% CI=35.5%, 80.4%) and 5 (48.6%; 95% CI=33.7%, 63.8%). NH other race/ethnicity youth who used e-cigarettes had the highest prevalence of using cannabis from Waves 1 (43.0%; 95% CI=28.4%, 59.1%) to 3 (59.0%; 95% CI=44.1%, 72.4%), whereas Hispanic youth had the highest prevalence in Wave 6 (46.9%; 95% CI=32.6%, 61.7%).

**Figure 4:**
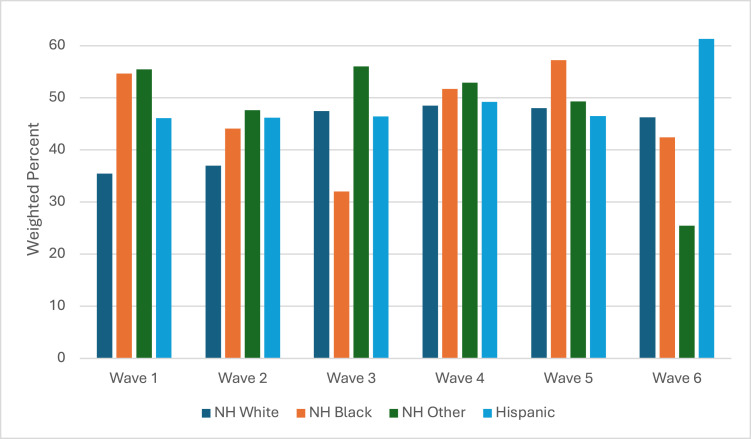
Racial/ethnic differences in cannabis-use prevalence among youth who use combustible tobacco. NH, non-Hispanic.

There were no significant racial/ethnic differences in cannabis use among youth who used combustible tobacco products in any wave (Figure 4 and Table 1). In Wave 5, NH Black youth who used combustible tobacco had the highest prevalence of using cannabis (57.2%; 95% CI=40.8%, 72.2%). NH Other youth who used combustible tobacco had the highest prevalence of using cannabis from Waves 1 (55.5%; 95% CI=41.3%, 68.8%) to 4 (52.9%; 95% CI=41.5%, 63.9%). In Wave 6, Hispanic youth who used combustible tobacco products had the highest prevalence of using cannabis (61.3%; 95% CI=35.7%, 81.9%).

## DISCUSSION

This study extends the literature by providing updated estimates of youth cannabis use among a nationally representative sample over a 9-year period (2013–2021). Cannabis-use prevalence among those using tobacco ranged from 36% to 61%. Changes in data collection methods and measures throughout the waves did not allow further analyses on specific types of and trends in cannabis use over time.^23^ However, multiple contextual factors may have impacted youth tobacco and cannabis use during this period. The tobacco and cannabis industries employ various strategies (e.g., appealing flavored products, social media marketing) to attract youth.^26^^,^^27^ Conversely, the enactment of protective public health policies (e.g., restricting sales of flavored vaping products,^28^ raising the federal minimum age for sale of tobacco products from 18 to 21 years^29^) and educational campaigns may have reduced youth’s ability to purchase tobacco products and increased youth’s awareness of harms related to tobacco and cannabis use. In addition, disruptions in product supply and social/peer engagement during the pandemic may have deterred youth from using tobacco and cannabis.^30^

Notably, this study indicated racial and ethnic disparities in cannabis use among youth who use e-cigarettes, particularly NH Black, NH other race/ethnicity, and Hispanic youth. Especially, Hispanic youth demonstrated the highest prevalence of cannabis use in Wave 6 (61.3% among those who used combustible tobacco and 46.9% among those who used e-cigarettes). Prior research suggests that although NH White youth are more likely to use e-cigarettes than other racial/ethnic groups,^31^ Hispanic/Latinx youth are likely to initiate e-cigarette use earlier in life.^32^ Varying cultural perceptions and social acceptances of tobacco and cannabis use across racial/ethnic groups may explain these disparities.^33^^,^^34^ Moreover, NH non-White and Hispanic/Latinx youth continue to be targets for tobacco^35^ and cannabis industry advertising,^36^ potentially exacerbating racial/ethnic disparities in substance use.

Tailored interventions are warranted to address racial and ethnic disparities in tobacco and cannabis use among youth. A review of culturally tailored prevention programs found that such interventions were associated with lower tobacco initiation rates among minoritized youth.^37^ These interventions may include adapting materials into the preferred language of the target population or incorporating culturally relevant visuals to enhance accessibility and engagement. Furthermore, educational campaigns that highlight the harms of tobacco and cannabis use, along with stricter regulations on product availability and marketing, may be effective in reducing substance use among youth more broadly.

### Limitations

Self-report data in this study may be subject to recall and social desirability biases because youth might underreport their tobacco and/or cannabis use owing to concerns about admitting access to products they are legally prohibited from obtaining. The authors were unable to disaggregate the NH other racial/ethnic category owing to the limited race classifications available in the public PATH data set. Future research should incorporate more granular racial/ethnic subgroup data to better understand patterns of tobacco and cannabis co-use and to inform culturally tailored interventions.

## CONCLUSIONS

Cannabis use is highly prevalent among youth who use tobacco, and racial and ethnic differences persist, with NH Black, NH Other, and Hispanic youth reporting the highest cannabis-use prevalence. Study findings highlight the urgent need for targeted interventions to address racial/ethnic disparities in cannabis and tobacco co-use and mitigate associated health harms among youth.

## Appendix Supplementary materials

Image, application 1

## Footnotes

Supplementary material associated with this article can be found in the online version at doi:10.1016/j.focus.2025.100392.
