# Supplementary material for: Racial and Ethnic Disparities in Cannabis Use Among U.S. Youth Who Use Tobacco: Findings From the Population Assessment of Tobacco and Health Study
Source: AJPM Focus. 2025 Jul 1;4(5):100392. doi: 10.1016/j.focus.2025.100392 (PMC12451367; doi:10.1016/j.focus.2025.100392)
Supplement: Supplementary file 1 [file mmc1.docx]

**Appendix Table 1. Number of participants for each race/ethnicity by wave**

| **Race/Ethnicity** | **Wave 1** | **Wave 2** | **Wave 3** | **Wave 4** | **Wave 5** | **Wave 6** | **Total** |
| --- | --- | --- | --- | --- | --- | --- | --- |
| **Non-Hispanic White** | 6,614 | 5,674 | 5,342 | 6,556 | 5,354 | 2,508 | 32,048 |
| **Non-Hispanic Black** | 1,859 | 1,577 | 1,518 | 1,912 | 1,480 | 635 | 8,981 |
| **Non-Hispanic Other** | 1,257 | 1,105 | 1,099 | 1,387 | 1,158 | 563 | 6,569 |
| **Hispanic/LatinX** | 3,920 | 3,502 | 3,444 | 4,374 | 3,522 | 1,626 | 203,88 |
| **Total** | 13,650 | 11,858 | 11,403 | 14,229 | 11,514 | 5,332 | 67,986 |

**Appendix Table 2. Prevalence of tobacco, cannabis, and co-use (Past 30-day use)**

| **Product use** | Total sample  **Weighted % (95 % CI)** | NH White  **Weighted % (95 % CI)** | NH Black  **Weighted % (95 % CI)** | NH Other  **Weighted % (95 % CI)** | Hispanic/Latinx  **Weighted % (95 % CI)** |
| --- | --- | --- | --- | --- | --- |
| Any tobacco use | 8.28 (7.83, 8.54) | 9.98 (9.47, 10.51) | 5.54 (4.77, 6.42) | 6.47 (5.79, 7.22) | 6.65 (6.10, 7.25) |
| Any use of E-cigarettes | 4.88 (4.62, 5.16) | 6.21 (5.81, 6.63) | 2.17 (1.77, 2.65) | 4.24 (3.70, 4.84) | 3.80 (3.39, 4.26) |
| Any use of combustible tobacco products | 4.36 (4.12, 4.62) | 5.10 (4.75, 5.48) | 3.86 (3.22, 4.63) | 3.33 (2.82, 3.93) | 3.70 (3.34, 4.11) |
| Any cannabis use | 5.96 (5.67, 6.26) | 6.18 (5.79, 6.59) | 5.96 (5.22, 6.78) | 5.35 (4.64, 6.17) | 5.91 (5.44, 6.42) |
| Exclusive patterns of tobacco and cannabis use |  |  |  |  |  |
| Tobacco use only | 5.32 (5.06, 5.60) | 6.76 (6.34, 7.20) | 3.26 (2.72, 3.92) | 3.83 (3.34, 4.39) | 4.14 (3.74, 4.59) |
| Cannabis use only | 3.06 (2.88, 3.26) | 2.86 (2.60, 3.13) | 3.61 (3.12, 4.19) | 2.71 (2.28, 3.22) | 3.45 (3.11, 3.83) |
| Co-use of tobacco and cannabis | 3.15 (2.96, 3.35) | 3.62 (3.35, 3.90) | 2.45 (2.04, 2.94) | 2.81 (2.30, 3.45) | 2.72 (2.40, 3.07) |

*Note.* Pooled values across six waves of data; NH = Non-Hispanic; CI = Confidence interval
